# Understanding inherent image features in CNN-based assessment of diabetic retinopathy

**DOI:** 10.1038/s41598-021-89225-0

**Published:** 2021-05-06

**Authors:** Roc Reguant, Søren Brunak, Sajib Saha

**Affiliations:** 1 Novo Nordisk Foundation Center for Protein Research, University of Copenhagen, 2200 Copenhagen N, Denmark; 2 Australian E-Health Research Centre, CSIRO, Perth, Australia

**Keywords:** Eye diseases, Machine learning

## Abstract

Diabetic retinopathy (DR) is a leading cause of blindness and affects millions of people throughout the world. Early detection and timely checkups are key to reduce the risk of blindness. Automated grading of DR is a cost-effective way to ensure early detection and timely checkups. Deep learning or more specifically convolutional neural network (CNN)—based methods produce state-of-the-art performance in DR detection. Whilst CNN based methods have been proposed, no comparisons have been done between the extracted image features and their clinical relevance. Here we first adopt a CNN visualization strategy to discover the inherent image features involved in the CNN’s decision-making process. Then, we critically analyze those features with respect to commonly known pathologies namely microaneurysms, hemorrhages and exudates, and other ocular components. We also critically analyze different CNNs by considering what image features they pick up during learning to predict and justify their clinical relevance. The experiments are executed on publicly available fundus datasets (EyePACS and DIARETDB1) achieving an accuracy of 89 ~ 95% with AUC, sensitivity and specificity of respectively 95 ~ 98%, 74 ~ 86%, and 93 ~ 97%, for disease level grading of DR. Whilst different CNNs produce consistent classification results, the rate of picked-up image features disagreement between models could be as high as 70%.

## Introduction

Diabetic retinopathy (DR) is a microvascular complication of both type 1 and type 2 diabetes mellitus, which causes abnormalities in the retina and is a leading cause of blindness in the world. About one third of diabetics have diabetic retinopathy and nearly all will eventually develop it. By 2030, DR is projected to affect 191 million people globally^1,2^. Although the visual impairment and blindness caused by diabetic retinopathy is preventable^3^, early detection is crucial^4^. To ensure early detection and timely treatment, current guidelines suggest that those with poorly controlled diabetes should be screened for DR at least once in a year^5^. Patients already diagnosed with DR need to be screened more frequently^6^. Screenings of DR typically involve capturing an image of the retina fundus, which is later assessed by expert ophthalmologists. With an increasing diabetic population, it is a challenge to provide specialist eye care to all. One of the major issues the public health system faces is the increasing waiting list for ophthalmology consultations. Automated grading for DR is the effective strategy to move forward.

Automatic grading for diabetic retinopathy has many benefits. It ensures reproducibility and increases efficiency and scalability while reducing access barriers such as costs, time, or availability. On top of that, machines have no subconscious biases nor subjectivity. For the same image, the algorithm will predict the same value every time.

Automatic grading for DR remained an active area of research for more than a decade now. Prior automated methods in DR grading rule-based machine learning approaches focused on feature engineering i.e. finding specific lesions that predict the grading^7,8^ which Calleja et al. reported at an accuracy of 97%, while Ravishankar et al. reached a sensitivity of 95.7% and a specificity of 94.2%. Using feature selection techniques, Nayak et al. achieved an accuracy of 93%, with a reported sensitivity of 90% and a specificity of 100%^9^. Different simple machine learning methods like support vector machines^10^ achieved 86% accuracy. With a sensitivity of 100% and a specificity of 53% Raychowdhury used k-nearest-neighbors and gaussian mixture models to select specific features and reduce the grading computation time to 3.4 s^11^. Casanova et al. compared logistic regression against random forests with the latter beating the 90% accuracy mark improving the former methods by 10%^12^. In the same manuscript, the authors show that logistic regression maintains a rather stable classification accuracy independently of the number of training samples. In 2012, Antal and Hajdu proposed a different approach^13^. Instead of creating an ensemble of classification methods, they created an ensemble of methods that propose regions likely to have lesions and based on that predict the grading. With this approach, they achieved 85% sensitivity and 90% specificity.

In recent years, there has been an increasing interest in applying deep learning (DL)-based techniques for automated grading of DR^14^. In comparison to conventional rule-based machine learning approach, which is based on pre-defined hand-crafted clinical features, DL learns those features by itself relying upon neural network architectures^15^. DL-based methods are in some cases found to outperform conventional rule-based methods with a wide margin^16–18^. Several promising DL-based methods in DR have been published^19–26^. Among these, Quellec et al., Esfahani et al., Jiang et al., Gulshan et al., and Liu et al. perform a binary classification predicting DR or not, whereas Abràmoff et al., Raman et al., and Zhang et al. trained models to predict multiple levels of DR. Quellec et al. used a pre-trained AlexNet with data augmentation and image preprocessing to achieve an area under the curve (AUC) in the Kaggle data set^27^ of 0.954. Esfahani et al. used ResNet34 on 35,000 images from the Kaggle data set to achieve 85% accuracy and 86% sensitivity. Jiang et al. classified referable DR and non-referable DR using three pretrained models (Inception-v3, Inception-ResNet-v2, and Resnet152) and concatenated the outputs with adaboost. They resized the 30,244 images of their own dataset to 520 × 520pixels which propitiated an accuracy of 88.6% and AUC of 0.946. Liu et al. opted for developing a custom-made model with 105 layers (WP-CNN) which outperformed ResNet, SeNet, and DenseNet with an accuracy of 90.84% in the publicly available STARE data set. For the multi-classification approaches, Abràmoff et al. predicted three different outcomes: no DR, referable DR, or vision threatening DR disregarding the five DR stages and grouping mild DR as no DR. They used a model ensemble using a random forest as an aggregator achieving an AUC of 0.98, a sensitivity of 96.8% and specificity of 87%. Pratt et al. developed a custom model to classify the images from the Kaggle dataset. The accuracy is 75% with a specificity of 95% and a sensitivity of 30%. Zhang et al. opted for a private dataset consisting of 13,767 images to train and evaluate the models. They used the pretrained architectures ResNet50, Inception-v3, Inception-ResNet-v2, Xception, and DenseNets. To concatenate the outputs they added fully convolutional layers on top of the models; thus achieving an accuracy of 96.5%, a specificity of 98.9% and a sensitivity of 98.1%.

Despite producing state-of-the-art performance, DL-based methods are often criticized for being a “black-box” or in other words for offering no explanation of how classification decisions are made. In Europe, General Data Protection Regulation—commonly known as GDPR—demands all algorithms to be able to potentially provide an explanation for the output^28^. In USA, the Defense Advanced Research Projects Agency published an announcement soliciting that artificial intelligence should be made explainable^29^.

Consequently, we aim to visualize the convolutional neural network (CNN) to explain its decision-making process in automated multi-stage DR grading. In this context, we adopt the Grad-cam visualization strategy^30^. We also develop a set of notions representing different ocular components in fundus to comprehend the inherent image features involved in the decision-making process. We critically analyze the inherent image features picked up by different CNNs and also compare that to human grading.

## Literature review

### Convolutional neural networks (CNNs)

Deep convolutional neural networks are typically presented as layers of interconnected “neurons” which exchange information. Data (e.g. an image) is fed into the network and “representations” of the data are then generated by each successive layer. For example, the first layer may represent the location and orientation of edges within an image, while successive layers may deal with higher levels of abstraction. Ultimately, output neurons are activated, and the data is classified. Typically, deep CNN architectures consist of several convolutional layers. Each convolutional layer has several convolutional filters that are applied on the image. Each convolutional layer at level *L* takes an image of dimensionality *dL* and applies *N* number of filters to produce *N* number of feature maps. This convolution operation produces an *N* dimensional image, one dimension per filter. This *N* dimensional image is then taken as input to the next convolutional layer at level *d*(*L* + 1). This process continues for several convolutional layers. Finally, several fully connected neural network layers are added on top of convolutional layers. Typically, the final layer consists of a soft-max classifier.

Since AlexNet in 2012^31^, CNN models have gained significant attention in image classification. In recent years, more advanced deep learning models have been developed such as Inception, ResNet, InceptionResNet, and Xception, which are reviewed in this section.

#### Inception

Inception models^32–34^ are types of CNNs that are highly optimized for image classification. The main difference between Inception and regular CNNs are the inception blocks. An inception block convolves the same input tensor with multiple filters and concatenates their results. In contrast, regular CNNs performs a single convolution operation on each tensor. Many versions (v1, v2, v3, etc.) of Inception are publicly available and improves upon the previous architectures. Inception-v3^34^ is one of the best performing models for image classification and is experimented in this work.

#### ResNet

ResNet is a type of CNNs with residual connection introduced by He et al.^35^. The authors argue that residual connections are inherently necessary for training very deep convolutional models, since residual connections help to overcome the vanishing gradient problem. ResNet50 is a schema of ResNet; one of the best performing nets and is used in this work.

#### InceptionResNet

InceptionResNet^36^ is the residual versions of the Inception networks. In addition with residual connections, 1 × 1 convolutions are required for the residual addition to work (they match the depth size). For example, in the case of InceptionResNet, batch-normalization is used only on top of the traditional layers, but not on top of the summations. To increase stability the residuals are scaled prior to adding them to the previous layer.

#### Xception

Xception^37^ is considered as the extreme version of Inception. The main feature is the depth-wise separable convolution. One benefit of the depth-wise separable convolution is that it does not require convolutions across all channels requiring fewer connections and producing an overall lighter model. Contrary to the inception architecture, Xception does not include intermediate ReLUs (non-linearities). Like ResNet and InceptionResNet residual connections are incorporated into the architecture.

### CNN Visualization methods

Visualization methods aim to produce “visual explanations” for decisions of CNN-based models to make them more transparent and explainable^38^. In order to build trust in intelligent systems a number of visualization methods have been proposed recently so that the input stimuli that excite CNNs could be made visually transparent^39^. A rudimentary approach in this category is the occlusion sensitivity method^40^. The method blocks—occludes—a region of the image and evaluates how much does the prediction change depending on the occluded region. The method quantifies the relative importance of the occluded regions, and thus identifies the region of interest in the image. Later, more advanced methods that did not require the modification of the image were introduced. The class activation mapping (CAM) method developed by Zhou et al. is an example to this category^41^. The method highlights the important regions of the image. Recently, advanced techniques that use the gradients to elucidate important regions in the input image have been proposed. SmoothGrad^42^ is one such method that highlights the pixels that have the most influence on the outcome by adding gaussian noise to each of the pixels over several copies of the same image and averaging the resulting gradients.

Very recently more advanced method named Grad-cam^30^, that combines the best from both CAM and gradient-based methods has been proposed. Grad-cam uses the backpropagation from the target outcome to highlight important and relevant areas for the prediction in the image and improves localization accuracy by using the gradients. It is one of most popular visualization methods for complex CNNs, and that is why is used in this study.

## Methodology

### Data sets

Retinal images available from two publicly available fundus datasets named EyePACS^27^ and DIARETDB1 were used in this study. EyePACS is the largest publicly available dataset and contains a total of 35,126 fundus images that were graded into 5 DR levels – No DR, mild NPDR, moderate NPDR, severe NPDR and proliferative DR. The grading ranges from 0 to 4, where 0 is the healthiest and 4 is the most severe state. The data were unbalanced and most of the fundus images were no DR. The number of images in each category were as follows no DR: 25806, mild NPDR: 2443, moderate NPDR: 5292, severe NPDR: 873 and proliferative DR: 708.

For the identification and classification of the lesions we used the ﻿DIARETDB1 dataset^43^. It was made public to enable evaluations and benchmarking of diabetic retinopathy retina fundus. It was labeled by four experts and it contains the well-known lesions: hard exudates (38), soft exudates (15), microaneurysms (39) and hemorrhages (39). It contains 89 images of which only 5 do not contain signs of diabetic retinopathy, 27 were classified as normal, 7 mild, 28 moderate and severe non-proliferative, and 27 proliferative. All images were used and no preprocessing was done other than resizing.

### Pre-processing and data augmentation

The images varied significantly in quality, dimensionality and aspect ratios. An expert manually visualized all images and removed the blurred, too dark or too bright images. Based on experiments on different image dimensionality that included 300 × 300, 512 × 512 and 700 × 700, we found that 512 × 512 is the best dimensionality that ensured overall top accuracy, sensitivity, specificity and area under the ROC. For the images with anon-symmetric aspect ratio, we cropped them with respect to their centers to make them square.

We split the data set into train (80%), validation (10%) and test (10%) sets.

We performed data augmentation specifically by rotation (in the range of 360 degrees), height and width shift (in the range 0.15), scaling (in the range 0.1) and flipping that added small variations to the training data in order to prevent overfitting. These small variations are randomly generated using ImageDataGenerator from TensorFlow each time the image is fetched.

To further improve the algorithm and decrease the risks of overfitting, we over- and undersampled the different classes. When more than 500 labeled images were present, the class was undersampled. When less than 500 labeled examples were present, the class was oversampled. In total, we had 500 images for each class. For the oversampled classes, some of the images may be repeated. Since all images are subjected to data augmentation, the probability to generate the same image is rare.

### Model development

Four state-of-the-art CNNs namely Inception-v3, ResNet50, InceptionresNet50 and Xception were independently trained to perform disease level grading of DR (No DR, mild NPDR, moderate NPDR, severe NPDR and proliferative DR). During the training process, we initialized the parameters of the CNN using transfer learning. More specifically, we used pre-trained models that were already trained using a very large image dataset named ImageNet44 to initialize the network parameters, which were then fine-tuned using the provided image dataset.

Glorot uniform initializer, also known as Xavier uniform initializer^44^ was used to initialize the weights of the new layers that were added on top of the base CNNs.

While training, we firstly trained the new layers for 5 epochs and then we trained the entire model for another 100 epochs with an early stopping callback with 20 epochs patience. We used the Adam optimizer^45^ with a learning rate of 0.001 and a cross entropy loss.

Once the models were trained, we used the Grad-cam (as a part of the explainability library tf-explain) for visual interpretation of the CNNs decision. For all models, we selected the last convolutional layer and the predicted label to generate the heatmaps.

## Results

### Reproducing state-of-the-art performance in CNN-based DR-level grading

**Figure 1:**
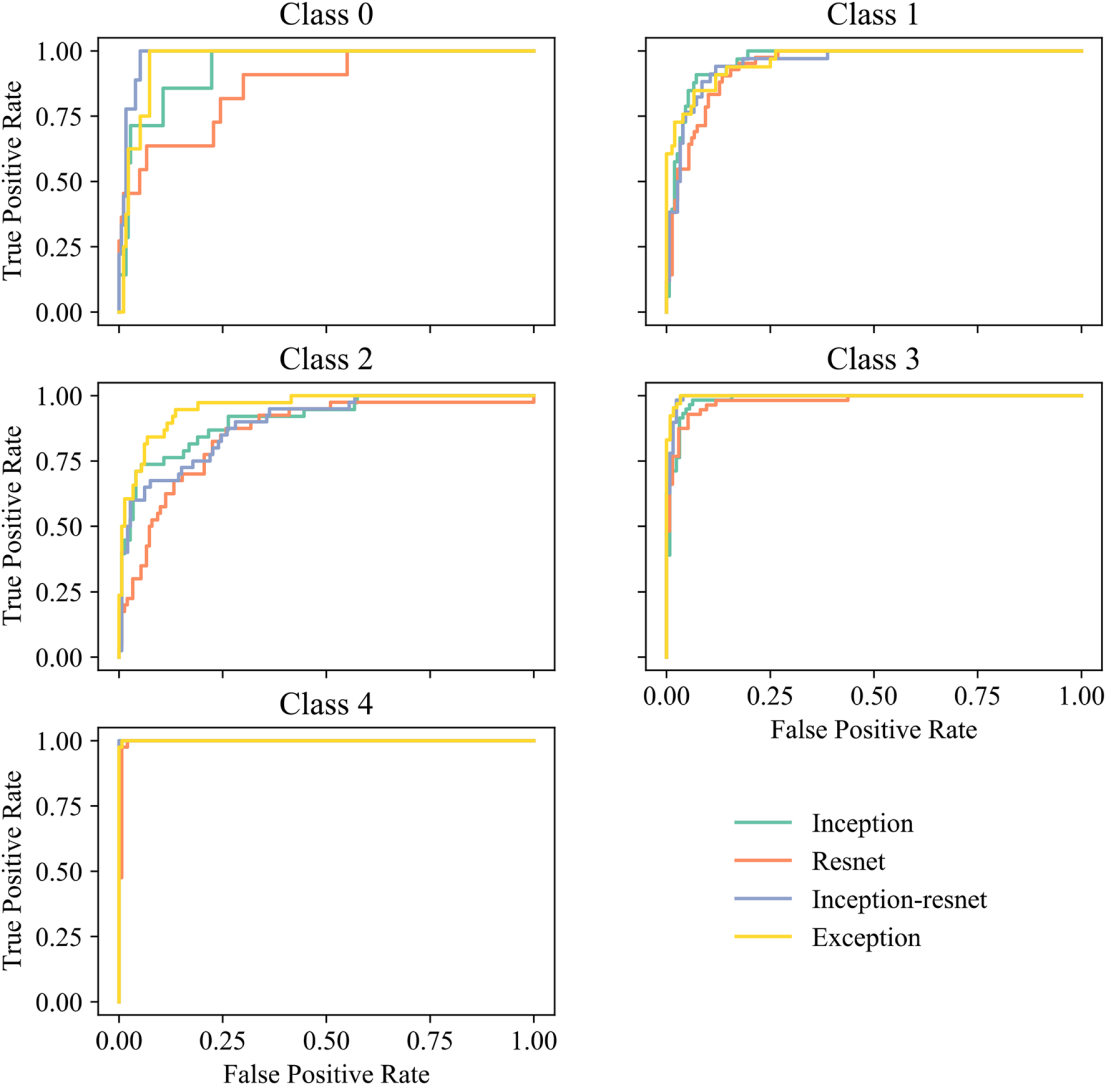
ROC curves for the models grouped by Class. (**a**) Class 0, (**b**) Class 1, (**c**) Class 2, (**d**) Class 3, (**e**) Class 4.

Figure 1 shows the ROC curves by the four different CNNs. Table 1 summarizes the accuracy, AUC, Kappa, Matthews coefficient, sensitivity (SE), specificity (SP), positive predictive value (PPV) and negative predictive value (NPV).

**Table 1:** Performance metrics for each of the models.

Model	Accuracy	AUC	Kappa	Matthews	SE	SP	PPV	NPV
Inception	0.94	0.96	0.80	0.81	0.81	0.95	0.73	0.96
Resnet50	0.89	0.95	0.62	0.62	0.73	0.93	0.68	0.94
Inception resnet	0.94	0.97	0.79	0.79	0.83	0.96	0.76	0.95
Xception	**0.95**	**0.98**	**0.83**	**0.83**	**0.86**	**0.96**	**0.87**	**0.98**

From Tables 1 and 2, we show that our models have comparable performance compared to those found in the literature. Although not fully comparable, Gulshan et al. showed an AUC of 0.99, sensitivity of 0.90 and specificity of 0.98. They did not split by gradings and a correct prediction was equal or worse grading than the true label. One prediction class was the aggregate of moderate, severe, or proliferative DR, while the other was severe, or proliferative DR. Thus, effectively removing the classes typically hard to classify. They also used several orders of magnitude more training data that improves their model generalizability. The best performing results with similar study design mentioned in the introduction had an AUC of 0.98 for the Abràmoff et al., a specificity of 98.9% and a sensitivity of 98.1% for Zhang et al.. In this report we show that our state-of-the-art CNNs range from a ROC AUC of 0.96 to a maximum of 0.98 matching the state of art published CNNs. Our models provide a wider range of sensitivity rangingfrom 73.5% up to 86.2%. With less variation the specificity ranges from 93.4% to 96.6%. Specificity and sensitivity are slightly inferior to the ensemble models described in previous literature. That was expected since ensemble methods benefit from exploiting the strengths of each individual model and producing an aggregated prediction at expense of other factors i.e. explainability. The models presented in the literature are typically developed with performance in mind. However, that is not the case for this manuscript where explainability is the end goal. Some of the method enhancement techniques, like model ensembling that previous literature used, are not compatible with pixel-wise feature visualization; thus impeding a proper visualization of the attention areas in the image.

**Table 2:** Sensitivity (SE) and specificity (SP) for each of the gradings.

Model	Grading 0		Grading 1		Grading 2		Grading 3		Grading 4	
	SE	SP	SE	SP	SE	SP	SE	SP	SE	SP
Inception	0.40	0.96	0.76	0.91	0.54	0.95	0.97	0.97	1.00	0.99
Resnet50	0.40	0.93	0.68	0.90	0.56	0.93	0.87	0.98	1.00	0.98
Inception resnet	0.70	0.94	0.68	0.93	0.54	0.96	1.00	0.96	1.00	1.00
Xception	0.30	0.99	0.94	0.94	0.77	0.97	1.00	0.98	1.00	0.99

Not all gradings are classified with the same performance. In Fig. 1, we show that grading zero has the worst classification performance. This is because the more subtle the features are the harder it is to correctly classify the image. In a healthy eye (grading zero) there are no specific features to detect, which is as well the reason why these images are often removed. Images with grading one improves significantly over grading zero because there are already features that allow the models to correctly predict some degree of lesions. Grading two gets slightly worse predictions than grading one because the models struggle to identify the severity of the lesions or if there is any lesion at all. Most of the incorrectly classified images for this class are split between grading zero and one. Grading three and four get almost perfect predictions because those lesions are severe and noticeable for both the doctors and the model. If there is a doctor disagreement, it is commonly on lower gradings^46^ which then may be reflected on the results. The model picks up the uncertainty on lower grades while showing robustness on higher grades.

### Visualizing and interpreting inherent image features picked-up by different CNNs

**Figure 2:**
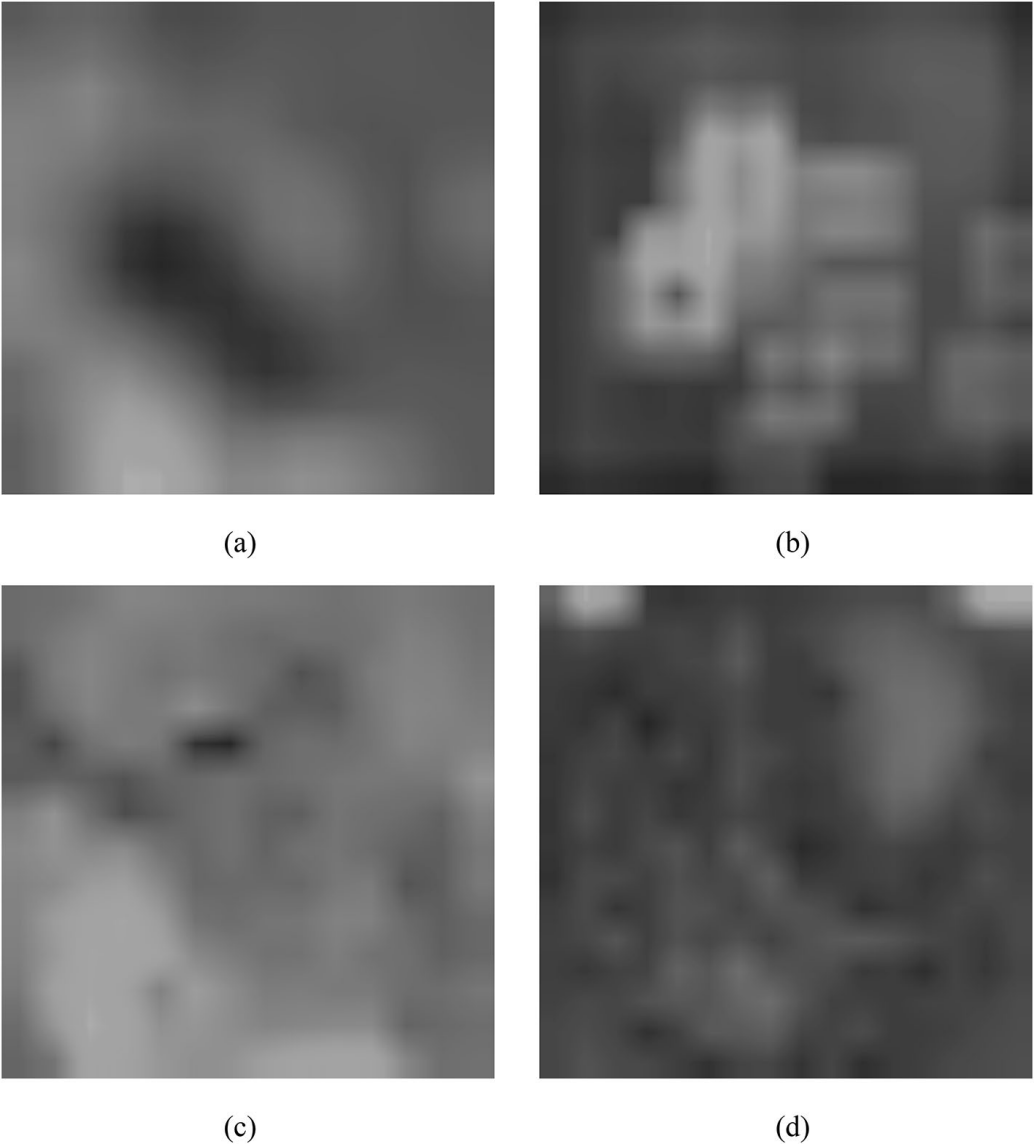
Sample heatmaps for (**a**) Inception, (**b**) ResNet, (**c**) InceptionResNet, (**d**) Xception.

**Figure 3:**
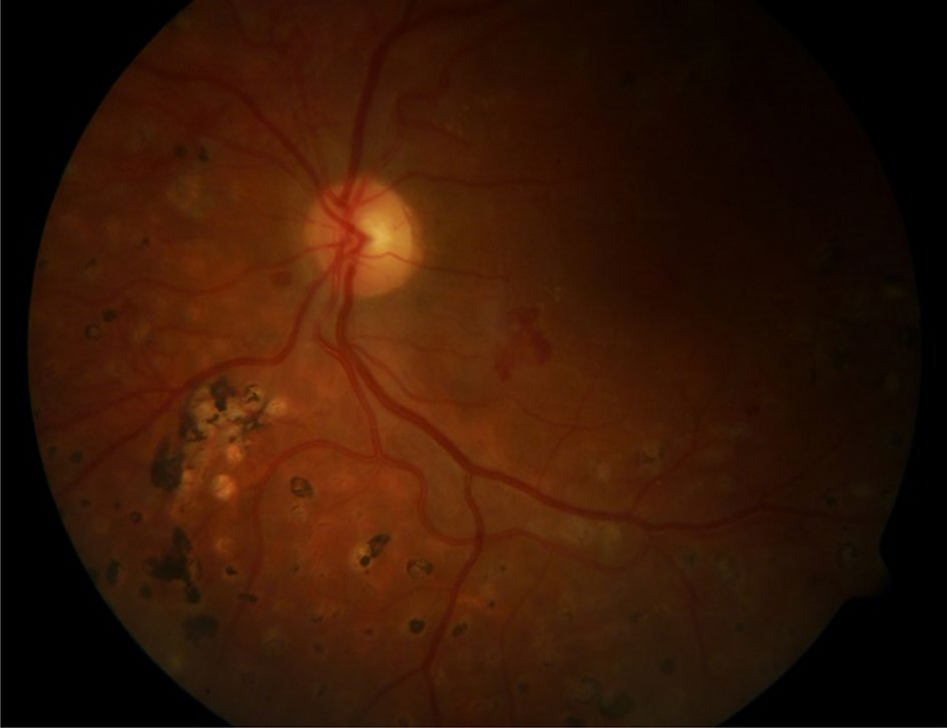
Example fundus image from DIARETDB1.

Figure 2 shows some sample heatmaps produced using Grad-cam for different CNNs for example fundus image shown in Fig. 3.

In order to interpret the information available in the visualization map and its clinical relevance, we compute the structural similarity of the visualization map with known pathology and other ocular components. We first binarize the heatmaps using OTSU’s method^47^ and then compute its structural similarity with known pathology and non-pathology components. Pathology components include microaneurysms, hemorrhages, and exudates. Non-pathology components include optic disc, vessels and other ocular regions in the fundus photograph except pathology components. Intersection over Union (IoU) scores^48^, defined below, are used to compute the structural similarity between the visualization map and ocular components. The IoU score for image *X*, visualization map *f* and component *c* is defined as:

1 $${IoU}_{X, f, c} = \frac{\left|{D}_{f}\left(X\right) \bigcap {M}_{c}(X)\right|}{\left|{D}_{f}\left(X\right) \bigcup {M}_{c}(X)\right|},$$

where ${M}_{c}\left(X\right)$ is the binary annotation mask of image *X* for component *c* (in ${M}_{c}\left(X\right)$, a pixel *p* is equal to 1 if *p* belongs to component *c*, and 0 otherwise).

A semi-automated system was developed to generate the ocular components. The system first produced the ocular components from the image using automated methods. A retinal image analysis expert then corrected the outputs.

**Figure 4:**
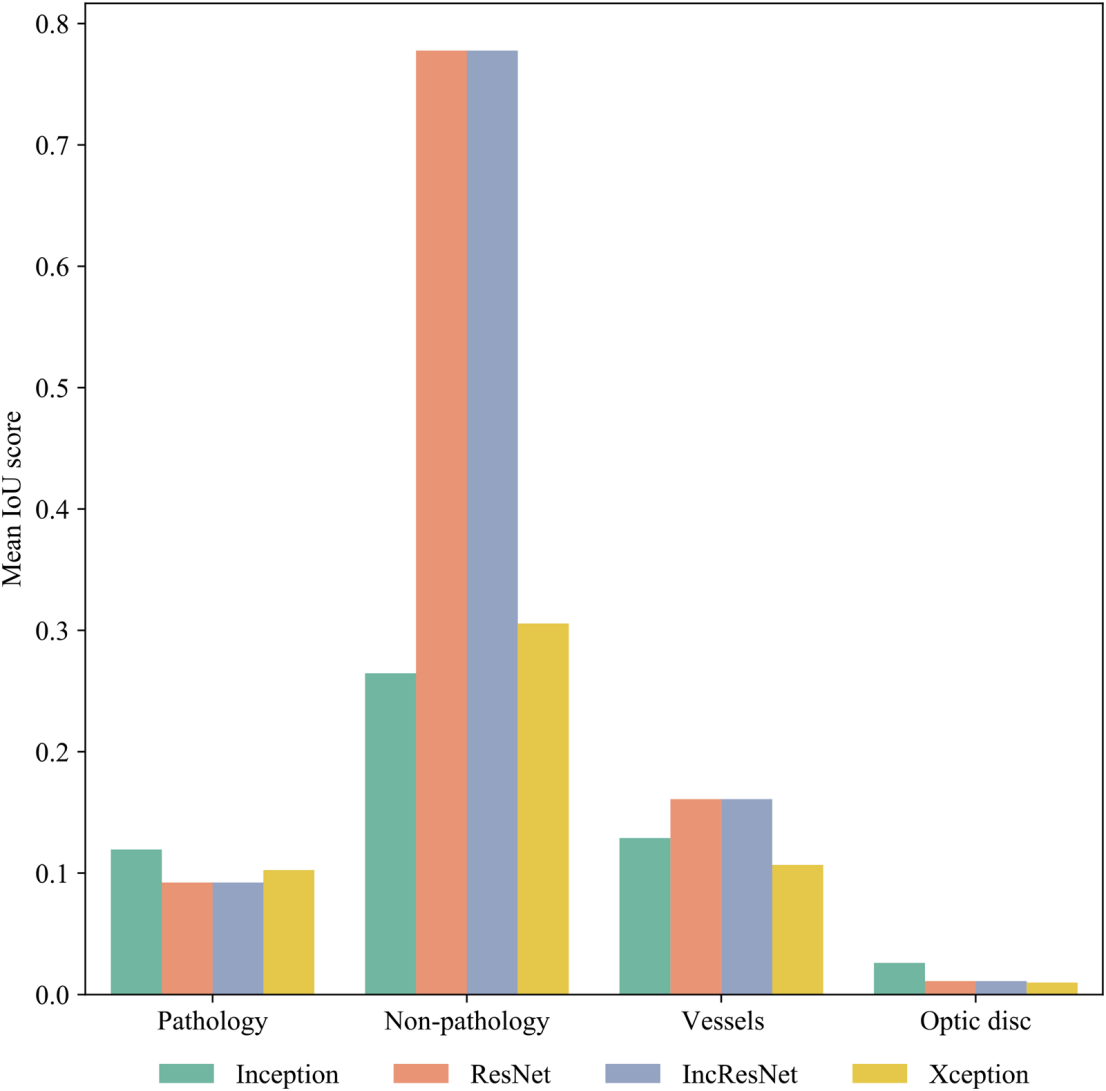
Plot of mean IoU scores, aggregated by ocular components.

Figure 4 shows the mean IoU scores for different CNNs.

From the results in Fig. 4, it is observable that in addition to the pathology regions, non-pathology regions are also playing important roles in the decision making of CNNs. In fact, as it is evident from the IoU scores, the non-pathology regions are way more dominant than the pathology regions when it comes to salient features picked-up by the CNNs. The IoU scores could be as high as 0.75 for non-pathology regions, whereas for pathology regions it is around 0.12. Greater diversity of IoU scores by different CNNs are apparent for non-pathology regions, which possibly explains many of these non-pathology features picked-up by the CNNs may not involve in the disease development. Among the non-pathology regions, vessels are the most contributing ocular components followed by optic disc. While experts’ assessments of DR using color fundus photographs principally focus on microaneurysms, hemorrhages, and exudates, recent studies in DR identifies some microvascular changes which are also associated with the DR development and progression^49^. Thus, identification of vessels by the CNNs as inherent image features in DR development and in addition with the consistency among different CNNs makes sense and create a basis for further investigation to identify more specific vascular patterns and features relevant to the disease development.

**Figure 5:**
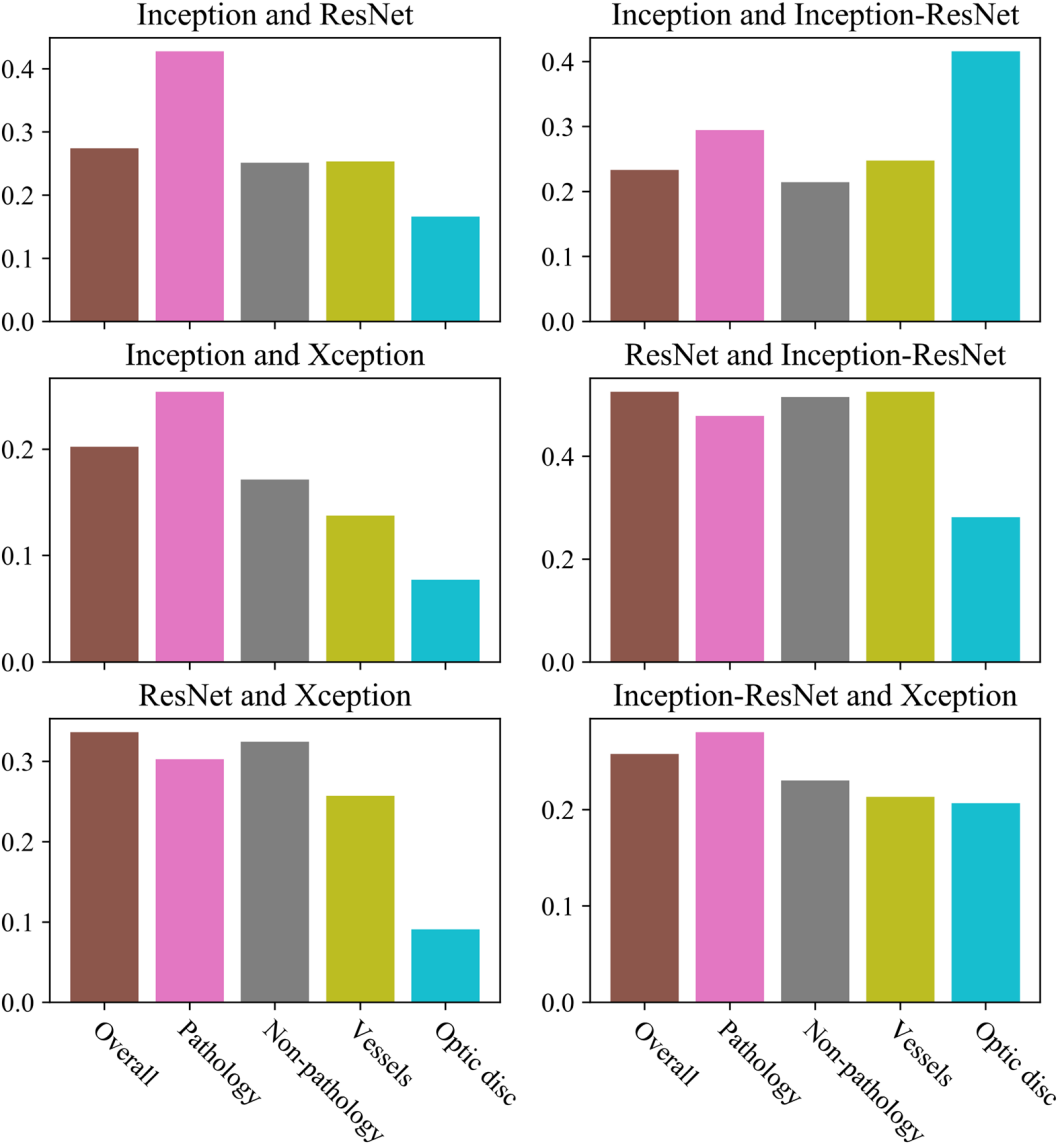
Mutual agreement of different CNNs when compared directly in the visualization map space.

Figure 5 shows the mutual agreement of different CNNs when analyzed in the visualization map space. It is observable that the pathologies are the areas where most overlapping is occurring across models. The inception models have the lowest overall overlapping focus regions compared to all the other models. With the lowest overlapping average of all combinations there is Inception and InceptionResNet. On the contrary, ResNet and InceptionResNet is the model combination with the highest overall overlapping regions, but also for all the specific features except the optic disc. Regarding pathological features, Inception is the model that clearly has the highest overlapping against all models. For non-pathologies the Xception model has an overlap of ~ 20% with the other models. The overlapping attention the models have on the vessels is in general slightly lower than the non-pathological features. Only with Inception and ResNet, and ResNet and InceptionResNet the vessels have higher overlapping regions compared to the non-pathologies. For the Inception and Resnet the models overlap is almost identical.

**Figure 6:**
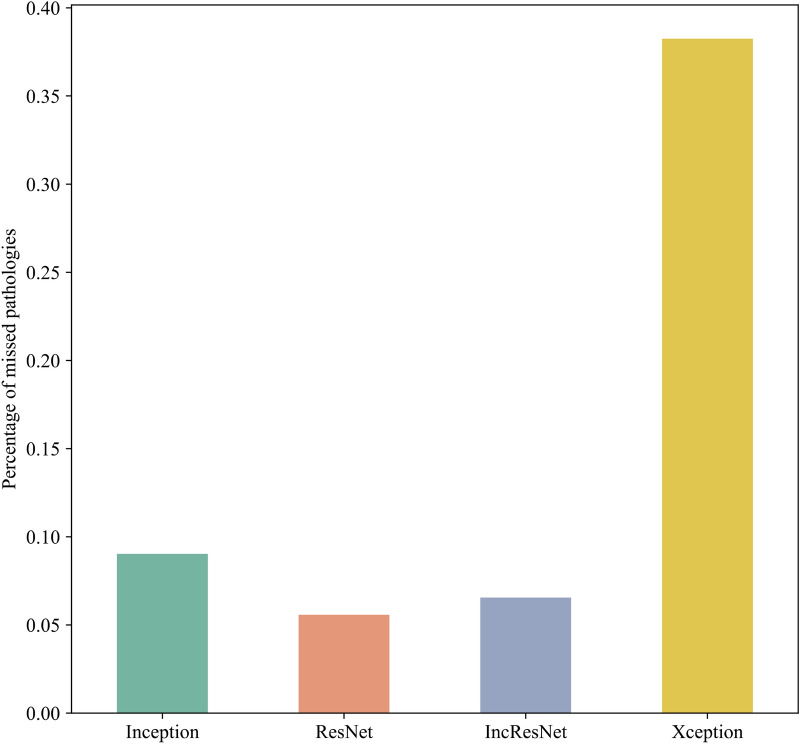
Overall DR pathology missed by different CNNs.

**Figure 7:**
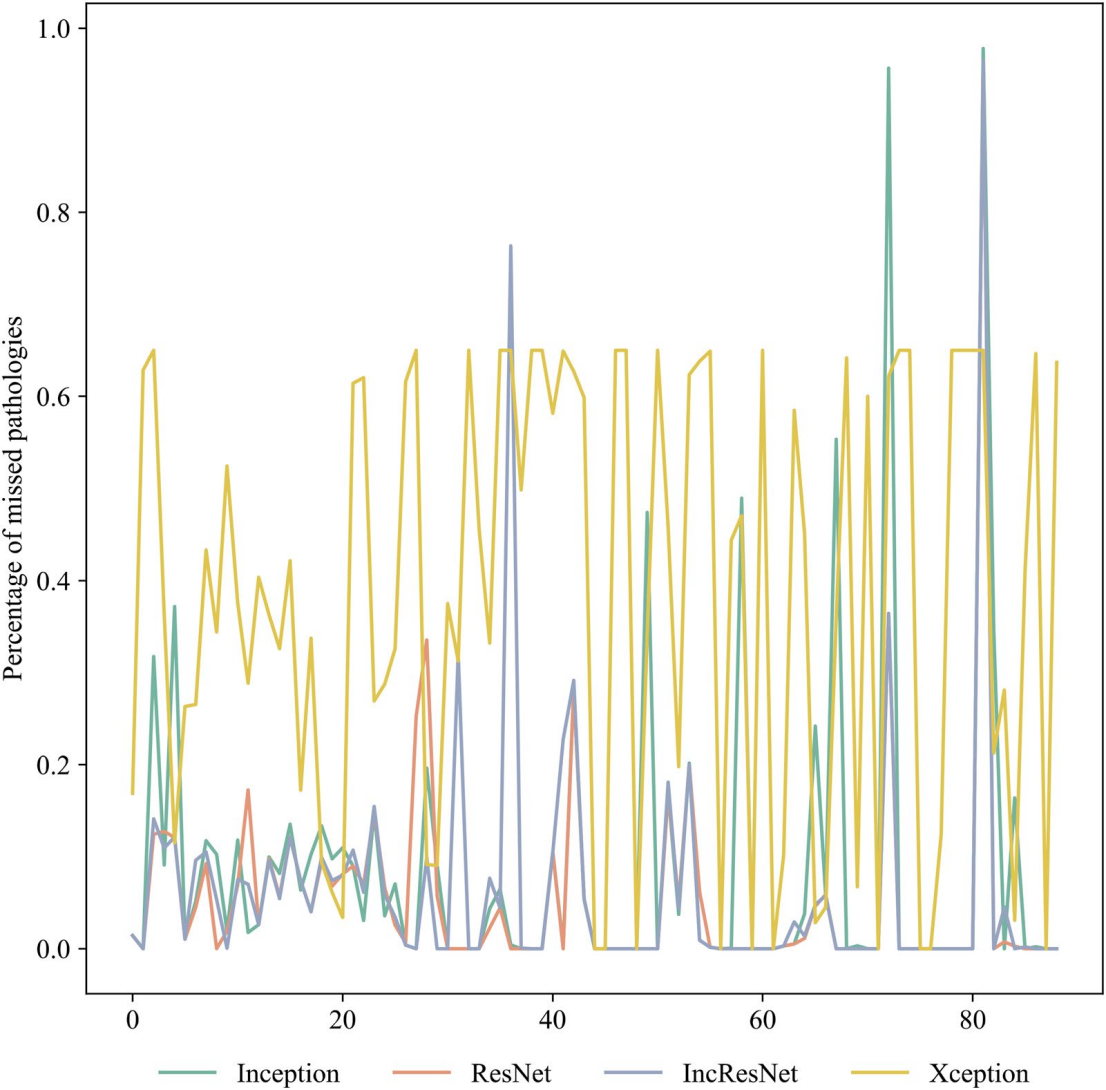
DR pathology missed by different CNNs on the test images.

Figures 6 and 7 summarize the percentage of DR pathology missed by different CNNs. Figure 6 shows the overall summary, whereas Fig. 7 details the DR pathology missed on the 89 test images.

From Figs. 6 and 7, it is observable that on average ResNet identifies the majority of the pathologies presented in the image with consistently low numbers on missed pathologies mostly under 20% and in any case below 40%. Similar results are seen for Inception-ResNet except for two conspicuous analysis where the missed pathologies arise above 60%. Inception occupies the third performance position with over 90% correctly identified pathologies. Except for one instance, the percentage of missed pathologies in each individual image do not overlap between inception and the previous two models. In general, Inception has a greater number of images with noticeable missed pathologies. With an average of more than 20 points difference Xception comes last with a seemingly consistent missed 60% of the pathologies for each of the images.

## Discussion

In this work, we show that CNNs can be used to predict diabetic retinopathy with high performance. Moreover, we clearly highlight that the use of deep learning and CNNs are able to focus and pay attention to the relevant parts of the images. Different models a varying different predictive power and feature selection capabilities, which it should not be seen as a limitation, but rather as an opportunity to increase our understanding of the models and the the results. Here, we have shown that deep learning models can be considered as fundamental and additional tools to support the decision-making process of clinicians. Without explicit lesion location information, the CNNs were able to identify most of the eye lesions that correspond to the grading.

CNNs can be successfully developed and trained on smaller datasets using transfer learning. Commonly used pre-trained models can reduce the number of training examples required while retaining state-of-art performances. Even though these models were pretrained and adjusted to fit the Kaggle data set, this publication has shown that the model predictions are generalizable and the models are able to maintain their predictive powers for different data sets. The ability to retain predictivity across data sets is especially important to deep learning models in order to show that the model is not overfitted and capable of similar performance in the future. This model’s performances could be improved with more images. However, the cost of adding new images has diminishing returns.

In this work, we have used high quality images, which is not always the case in the real-world scenario. The proposed tool does not assess the quality of the images. So, when a lower quality image is present, a grading will be provided leading to erroneous classification. To solve this problem, several tools have been developed to assess the quality of the images and recommend the healthcare workers to take another image^50,51^.

The strengths of this work are several. We were able to train several CNN models for disease level grading of DR using available datasets. Within this context we adopted a CNN visualization strategy that enabled to critically analyze the grading for the different images and models.

A limitation of this study is that the training and validation datasets are limited. Although we used transfer learning to enhance model performance, limited data forces the models to rely more heavily on the pretrained weights. Larger datasets can potentially increase the model performance. Furthermore, neither EyePACS nor DIARETDB1 datasets have specific information available about diabetic macular edema (DME) or laser photocoagulation scars. Therefore they were not assessed in this study.

Although no modifications of the model were required a priori, there were some model architecture constraints. The explainability methods required CNN layers to be close to the head of the model; thus, removing the possibility of ensembling different CNN models. This limitation may have hurt slightly the end performance at expense of the explainability. Other explainability tools require much larger modifications and even posterior training, which we consider a considerable deviation of the original purpose of this manuscript. Although we used state of art techniques, future deep learning models are likely to learn and extract features from fewer images with higher performances.

The models were trained with images and outcome labels without explicit definitions of the predictions. However, the network learned the features that were critical for the correct prediction, potentially unknown or ignored by human inspection.

## Conclusions

In this paper, we showed that CNN based methods are able to produce state-of-the-art performance in DR detection. We applied a CNN visualization strategy to discover the inherent image features involved in the disease grade prediction. We then later rationalized those features with respect to expert’s knowledge. We also critically analyze different CNNs by taking into account what features were considered in determining the disease status and justify their clinical relevance. Three out of the four models identified over 90% of the features presented in the images.

With ascending trends of diabetes in all ages worldwide, diabetic retinopathy is one of the comorbidities that can be easily monitored and automated at low cost. It is vital that DL enters the clinics and alleviates the workload of the professionals working in the sector in addition to provide higher accuracy, efficiency, and reproducibility of the diagnosis. However, it is crucial that doctors and patients are aware of the decision-making process of the AI in order to support the diagnosis and consider further clinical paths.

Our best performing model—the Xception model—achieved a 95% accuracy with a sensitivity of 86% and specificity of 96% thus being consistent with the state of art models. This produces a competitive good performance for a set of models whose targets were interpretability and not performance optimization.

We have shown that even though CNN models have not been trained to identify lesions in the retina fundus images, models intuitively do so. Previous studies focus on the model performance without considering the relevant features while our analysis shows that deep learning models are capable of understanding and explaining the features selected.
